# Efficient inhibition of African swine fever virus replication by CRISPR/Cas9 targeting of the viral p30 gene (CP204L)

**DOI:** 10.1038/s41598-018-19626-1

**Published:** 2018-01-23

**Authors:** Alexandra Hübner, Bjoern Petersen, Günther M. Keil, Heiner Niemann, Thomas C. Mettenleiter, Walter Fuchs

**Affiliations:** 1 Institute of Molecular Virology and Cell Biology, Friedrich-Loeffler-Institut, Federal Research Institute for Animal Health, 17493 Greifswald-Insel Riems, Germany; 2 Institute of Farm Animal Genetics, Friedrich-Loeffler-Institut, Federal Research Institute for Animal Health, 31535 Neustadt, Germany

## Abstract

African swine fever is a devastating viral disease of domestic and wild pigs against which no vaccine or therapy is available. Therefore, we applied the CRISPR (clustered regularly interspaced short palindromic repeats) – Cas9 nuclease system to target the double-stranded DNA genome of *African swine fever virus* (ASFV). To this end, a permissive wild boar lung (WSL) cell line was modified by stable transfection with a plasmid encoding Cas9 and a guide RNA targeting codons 71 to 78 of the phosphoprotein p30 gene (CP204L) of ASFV. Due to targeted Cas9 cleavage of the virus genome, plaque formation of ASFV was completely abrogated and virus yields were reduced by four orders of magnitude. The specificity of these effects could be demonstrated by using a natural ASFV isolate and escape mutants possessing nucleotide exchanges within the target sequence, which were not inhibited in the Cas9-expressing cell line. Growth of the cell line was not affected by transgene expression which, as well as virus inhibition, proved to be stable over at least 50 passages. Thus, CRISPR-Cas9 mediated targeting of the ASFV p30 gene is a valid strategy to convey resistance against ASF infection, which may also be applied in its natural animal host.

## Introduction

African swine fever (ASF) is an economically important infectious disease of swine which causes mortality rates of up to 100% in domestic pigs and wild boar. In contrast, infections of African wild pig species (warthogs and bush pigs) are mostly subclinical. Whereas ASF is endemic in Sub-Saharan Africa, previous outbreaks in other parts of the world like South America and Southern Europe could be eliminated, except on the island of Sardinia. However, in 2007 the virus was introduced from Africa to the Caucasian countries Georgia and Armenia. From there it spread via the Russian Federation, Ukraine, and Belarus to the eastern part of the European Union, namely the Baltic states, Poland^1,2^ and, very recently, the Czech Republic and Romania^3^.

The causative agent of the disease, African swine fever virus (ASFV), represents the hitherto sole member of the family *Asfarviridae*^4^. ASFV is a large (approx. 200 nm) enveloped virus with an icosahedral capsid and two membranes at its inner and outer sides. The 170 to 193 kbp double-stranded DNA genome contains between 150 and 167 open reading frames depending on the isolate, and is predominantly replicated in virus factories located in the cytoplasm of infected cells^5^. Porcine macrophages are the major natural host cells of ASFV^6^. However, unlike other known DNA viruses of mammals, it also replicates in several soft tick species^7^. Ticks play an important role for virus transmission between wild pigs in Africa, but not in Central Europe, where permissive arthropod vectors are absent, and where movement of infected pigs and pig products is the main cause for spread^8,9^.

Up to now, no efficacious vaccines against ASFV infection are available, although recent attempts to generate e.g. DNA vaccines^10^, vectored vaccines^11^, or live attenuated vaccines by defined gene deletions^12,13^ yielded promising results in laboratory experiments. However, in most cases the considerable variability of the ASFV genome and deduced proteins^5^ limits the efficacy of potential vaccines, although cross-protection against different ASFV genotypes has been reported for several live vaccine candidates^14^. Furthermore, several potential antiviral drugs have been evaluated^1^, and inhibition of ASFV gene expression and replication by specific small interfering RNAs has been shown *in vitro*^15,16^.

During recent years, efficient RNA-mediated prokaryotic defence systems like the clustered regularly interspaced short palindromic repeats (CRISPR) - Cas9 nuclease system of *Streptococcus pyogenes*^17,18^ have been converted into powerful tools for genome editing in eukaryotes^19–21^. This also opens new possibilities for prevention and control of virus infections^22^, i.e. by facilitating targeted viral gene deletions or mutations during development of attenuated live vaccines. On the other hand, resistant host organisms can be generated either by knock-out of cellular virus receptor genes, or by constitutive expression of antiviral RNAs by the host. CRISPR/Cas9 mediated receptor targeting has been successful in rendering cells resistant against human immunodeficiency virus (HIV-1) infection *in vitro*^23,24^, and swine against the porcine respiratory and reproductive syndrome virus *in vivo*^25,26^. However, these pigs were not resistant against ASFV, although the knocked-out macrophage surface protein CD163 has also been considered as a putative ASFV receptor^27^. Direct targeting of viral genes has been shown to confer virus resistance in plants and insects^28,29^. Since breeding of clonal transgenic swine modified by using the CRISPR/Cas9 system has been repeatedly shown to be feasible^30,31^, we decided to attempt the development of ASFV-resistance using this technology.

## Results

### Generation of CRISPR/Cas9 cell lines

To identify and clone suitable CRISPR/Cas9 target sequences of the ASFV genome we used the vector plasmid pX330-U6-Chimeric_BB-CBh-hSpCas9^20^ which has been originally designed for editing of mammalian genomes, and, therefore, encodes nuclear localization signals (NLS) at both ends of the Cas9 open reading frame (ORF). Since ASFV replicates predominantly in the cytoplasm of infected cells^5^, these signals were removed. In similar approaches, NLS-less Cas9 has been used for successful targeted mutagenesis of vaccinia virus, which also replicates in the cytoplasm^32^. Furthermore, a neomycin resistance gene was inserted to enable the isolation of stable CRISPR/Cas9 cell lines.

**Figure 1:**
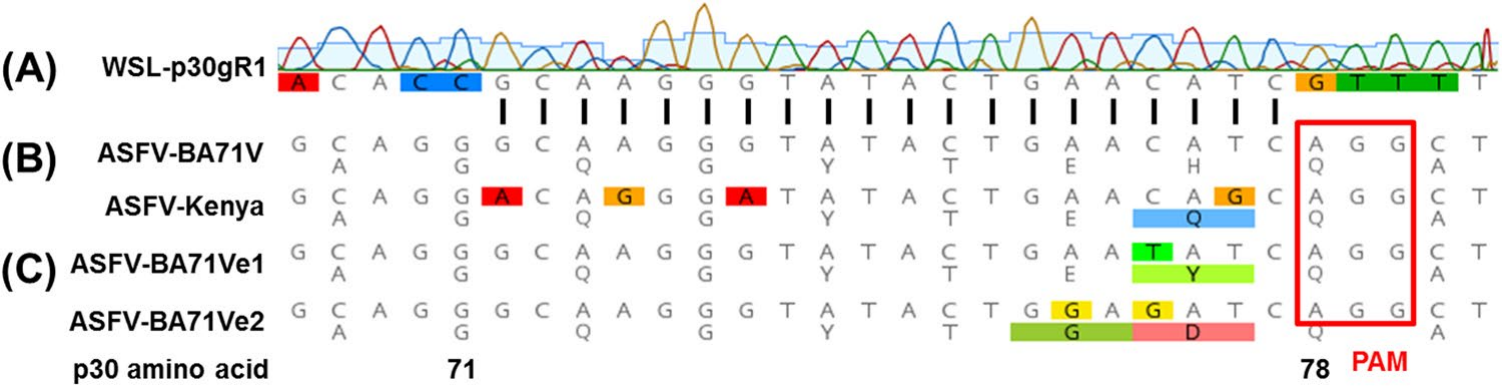
Sequence comparison between the ASFV p30 gene-specific guide RNA gene sequence of WSL-gRp30 cells **(A)**, the corresponding viral sequences of ASFV-BA71 and ASFV-Kenya1033 **(B)**, and of two escape mutants of ASFV-Ba71VΔTKdsRed isolated after passage on WSL-gRp30 cells **(C)**. A chromatogram indicating nucleotide peaks and quality is shown above the excerpt of the determined cellular sequence, and the deduced p30 amino acid sequences with position numbers are given below the viral gene fragments. Differences to ASFV-BA71 are coloured, and the targeted 20 nt (vertical lines) as well as the following PAM (red rectangle) are indicated.

**Figure 2:**
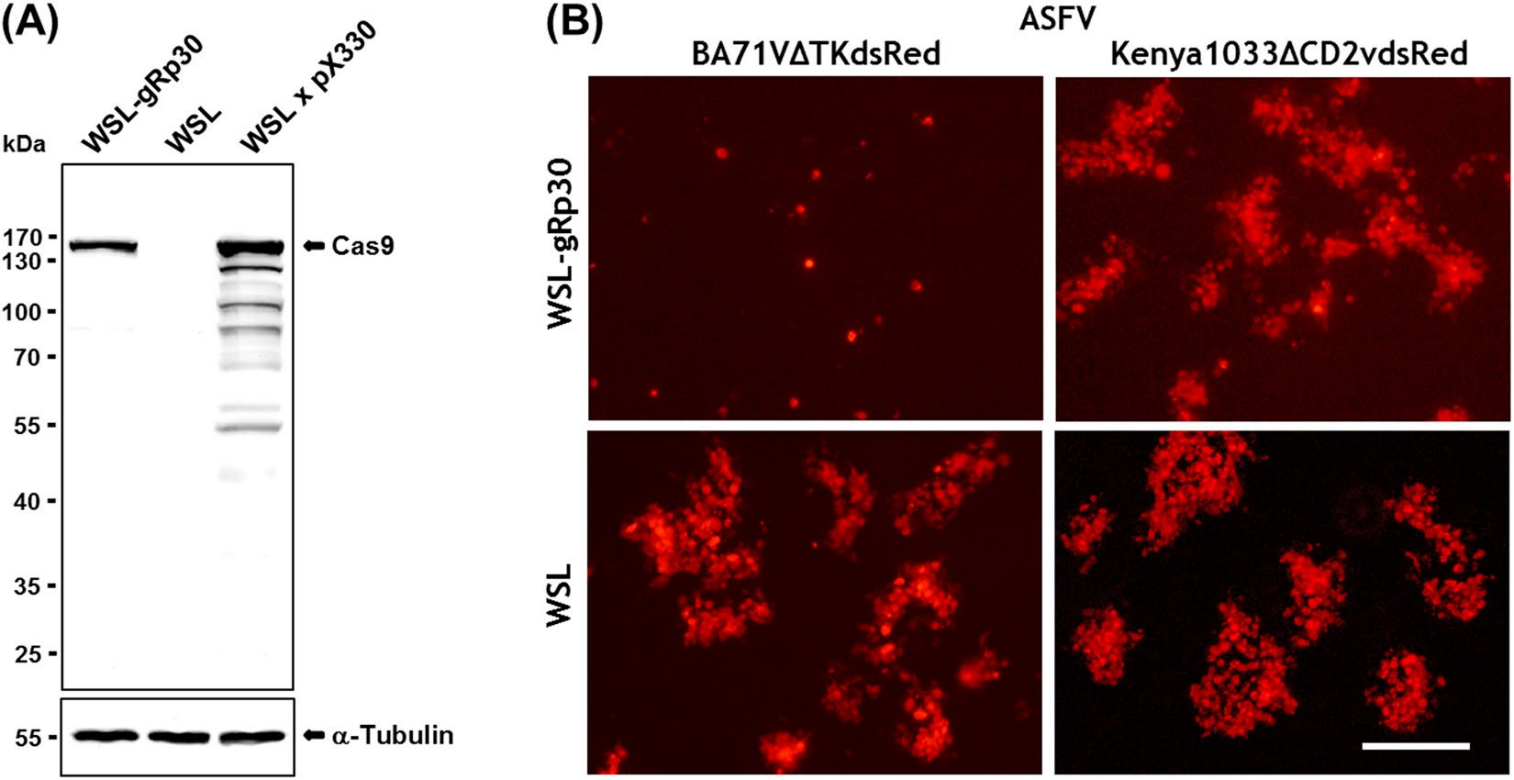
**(A)** Expression of FLAG-tagged Cas9 in WSL-gRp30 cells, and in WSL cells transfected with pX330-ΔNLS1/2neoR was detected by Western blot analyses using an anti-FLAG monoclonal antibody. The expected 161.3 kDa protein is indicated by an arrow. Additional bands detected in transiently expressing cells presumably represent degradation products of Cas9. A parallel blot incubated with an α-tubulin specific monoclonal antibody served as loading control. Molecular masses of marker proteins are indicated. **(B)** Microscopic fluorescence images showing dsRed-expressing single cells or growing foci and plaques on WSL-gRp30 and WSL cell monolayers of comparable densities at day 5 after infection with the same dilutions of ASFV-BA71VΔTKdsRed or ASFV-Kenya1033ΔCD2vdsRed. Bar indicates 200 µm.

Into the resulting vector pX330-ΔNLS1/2neoR several guide RNA sequences targeting the ORFs of the major capsid protein p72 (B646L), and the viral DNA polymerase (G1211R) of ASFV were inserted at the 5′-end of the trans-activating CRISPR RNA (tracrRNA) gene, but exerted only minor effects on virus replication (not shown). Possibly, the affinity of the Cas9 nuclease to these target sites was suboptimal, or they were highly tolerant against in-frame deletions or insertions resulting from non-homologous end joining. The finally chosen CRISPR/Cas9 guide RNA target sequence (Fig. 1A) encompassed codons 71 to 78 of the ORF encoding the highly immunogenic phosphoprotein p30 (CP204L) of the completely sequenced ASFV strain BA71V (GenBank accession NC_001659^33^). However, this target sequence is also conserved in the p30 gene of most other ASFV isolates of different genotypes including the viruses currently circulating in Eastern Europe^34^. Within the viral genome the target sequence was followed by the protospacer adjacent motif (PAM) 5′-NGG-3′, which is required for binding of and cleavage by the Cas9 nuclease of *Streptococcus pyogenes* (Fig. 1). The corresponding expression plasmid was used for transfection of an ASFV-permissive wild boar lung cell line (WSL), and the obtained neomycin-resistant cell clones were tested by Western blotting for Cas9 expression (Fig. 2A), and for presence of the target specific guide RNA sequences by PCR amplification and sequencing of genomic DNA. Our studies demonstrated that in several cell clones the nuclease and the p30-specific gRNA were stably expressed over many (>50) passages. Nevertheless these cells exhibited similar growth as the parental line, indicating that deleterious off-target reactions of Cas9 nuclease did not occur. This was not surprising, since no sequences matching the chosen gRNA sufficiently^21^ could be detected within the porcine genome.

### ASFV replication in CRISPR/Cas9 cells

To facilitate infection studies, two ASFV recombinants containing expression cassettes for the red fluorescent protein dsRed were used. The first one, ASFV-BA71VΔTKdsRed, was derived from the cell culture-adapted strain BA71V^33^ by substitution of the viral thymidine kinase (TK) gene (K196R) by a dsRed expression cassette as described previously^35,36^. The second mutant, ASFV-Kenya1033ΔCD2vdsRed, was derived from the isolate ASFV-Kenya1033 (publication in preparation) by a similar substitution of the CD2v ORF (EP402R). The CP204L ORF of the Kenyan virus exhibited 87.1% nucleotide sequence identity to that of ASFV-BA71V, resulting in 85.1% identity of the deduced amino acid sequences. However, within the chosen 20 nucleotide (nt) target sequence the Kenyan virus showed 4 base exchanges compared to most other characterised ASFV isolates (Fig. 1B). Remarkably, only one of these alterations led to an amino acid substitution of histidine (H) by glutamine (Q) at position 77 of p30. Because of these mismatches, binding of the guide RNA, and subsequent Cas9 cleavage of the ASFV-Kenya1033 genome appeared unlikely^21^.

**Figure 3:**
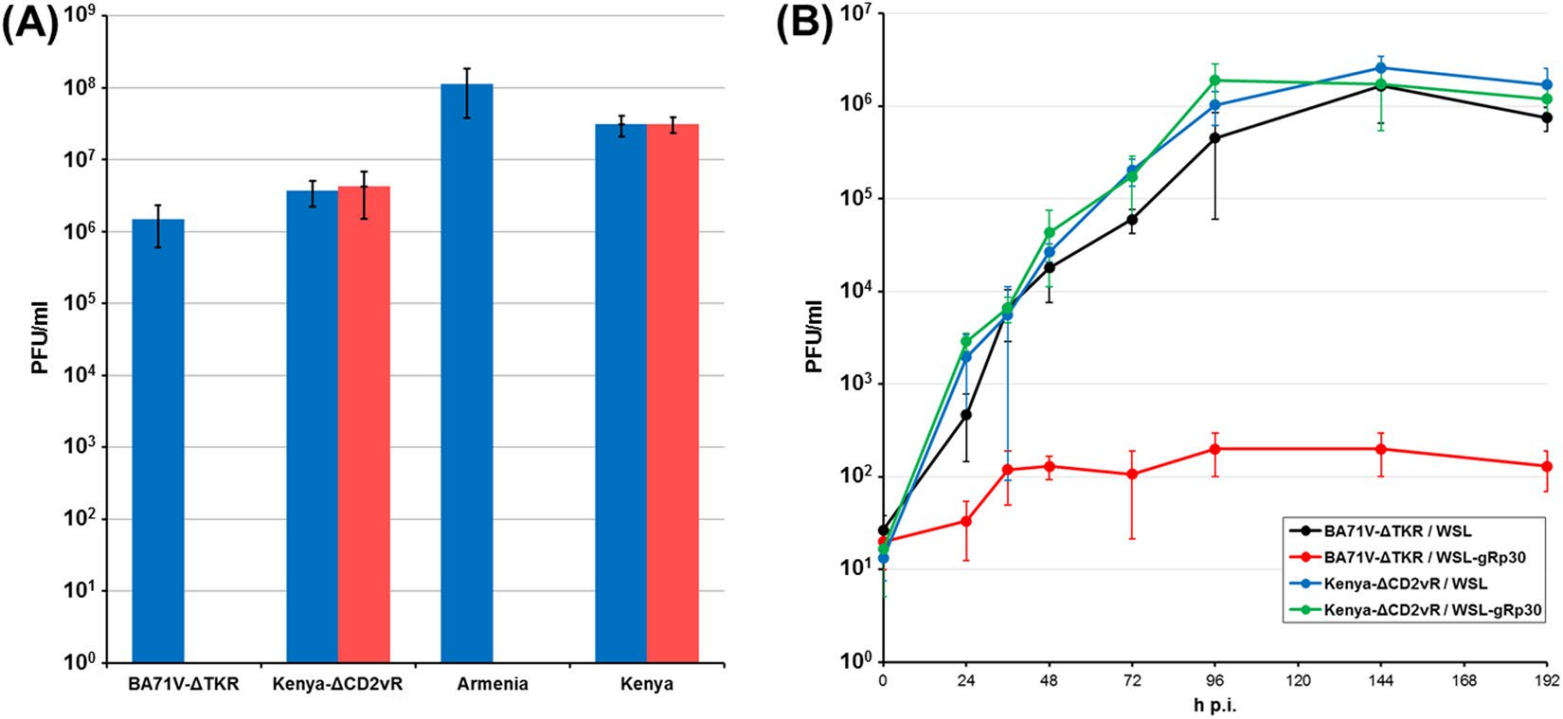
Differential ASFV replication in WSL-gRp30 cells. **(A)** Plating efficiencies of ASFV-BA71VΔTKdsRed, ASFV-Armenia, ASFV-Kenya1033ΔCD2vdsRed, and ASFV-Kenya1033 were analysed in parallel on WSL (blue bars) and WSL-gRp30 cells (red bars). Mean values of the infectious virus titres (PFU/ml) determined in three experiments and standard deviations are indicated. **(B)** Growth kinetics of ASFV-BA71VΔTKdsRed and ASFV-Kenya 1033ΔCD2vdsRed on WSL and WSL-gRp30 cells after synchronized infection at an MOI of 0.03. Shown are the mean virus titres at indicated times after infection (h p.i.) and standard deviations of three experiments.

Different Cas9 expressing WSL-gRp30 cell clones and parental WSL cells were infected in parallel with serial dilutions of ASFV-BA71VΔTKdsRed and ASFV-Kenya1033ΔCD2vdsRed. Obviously, both viruses were able to enter all cell lines and to initiate viral gene expression, since single red fluorescent cells became visible after 2 to 3 days. Since dsRed expression was under control of the late p72 promoter of ASFV^37,38^, viral DNA replication also seems to take place in all tested cell lines. However, plaque formation of ASFV-BA71VΔTKdsRed was impaired in several of the transgenic cell clones. One cell line exhibiting an almost complete inhibition of the spread of ASFV-BA71VΔTKdsRed, but permitted efficient plaque formation of ASFV-Kenya1033ΔCD2vdsRed (Fig. 2B, Fig. 3A), was investigated in more detail.

Since BA71V^33^ is a highly *in vitro* passaged, avirulent ASFV strain, we also tested a virulent Armenian ASFV isolate from 2007, and the unmodified isolate ASFV-Kenya1033, which were both passaged on WSL cells only few (≤10) times. As expected, plaque assays revealed that replication and spread of ASFV-Armenia, which possesses an identical target sequence to BA71V, were severely inhibited on WSL-gRp30 cells, whereas ASFV-Kenya1033 showed similar replication properties on WSL-gRp30 and WSL cells (Figs 3A, S1).

Furthermore, WSL-gRp30 and normal WSL cells grown in microtiter plates were infected at a multiplicity (MOI) of 0.03 plaque forming units (PFU) per cell of ASFV-BA71VΔTKdsRed or ASFV-Kenya1033ΔCD2vdsRed and incubated for various periods of time at 37 °C. Titration of virus progenies in total cell lysates revealed that both ASFV recombinants exhibited similar replication kinetics on native cells leading to maximum titres of approx. 2 × 10^6^ PFU/ml after 96 h (Fig. 3B). However, on WSL-gRp30 cells titres of ASFV-BA71VΔTKdsRed increased only tenfold compared to the input level, and did not exceed 2 × 10^2^ PFU/ml during the course of the experiment (Fig. 3B). In contrast, replication of ASFV-Kenya1033ΔCD2vdsRed was unaffected on WSL-gRp30 cells (Fig. 3B). One step growth kinetic studies (MOI 3) led to comparable results (not shown).

The almost complete inhibition of spread and productive replication of ASFV-BA71VΔTKdsRed and ASFV-Armenia on WSL-gRp30 cells was considered to be due to specific inhibition by CRISPR/Cas9-mediated cleavage of the virus genome at the p30 gene locus. The secreted and membrane-associated phosphoprotein p30 has been shown to be involved in host cell attachment and entry of ASFV^39–41^, and up to now no corresponding gene deletion mutant could be generated (unpublished results), which strongly indicated that p30 is indispensable for replication of ASFV. The essential function of p30 was confirmed by the present study.

Interestingly, comparative Western blot analyses of lysates of WSL and WSL-gRp30 cells which had been infected with ASFV at a MOI of 5 did not reveal an apparent reduction of the level of p30 caused by CRISPR/Cas9 targeting of CP204L (Fig. S2). This might be due to the very abundant expression of p30 at early times after infection^39,40^ under control of a strong promoter independent of viral DNA replication^42^. In contrast, expression of the major capsid protein p72, which is under control of a true late promoter^37,38,42^ was specifically reduced in WSL-gRp30 cells infected with sensitive ASFV variants (Fig. S2).

### Characterization of resistant virus mutants

CRISPR/Cas9 targeting of essential genes in other, e.g. herpesviruses or HIV-1 genomes led to the occurrence of escape mutants resulting from small in-frame deletions after repair of the cleaved DNA, or from proliferation of spontaneous virus variants, which are no longer recognised by the specific guide RNAs^43,44^. In contrast, consecutive (>10fold) passages of ASFV-BA71VΔTKdsRed at low dilutions on WSL-gRp30 cells or repeated splitting of the abortively infected cells did not lead to enrichment of virus variants capable of more efficient replication in 8 of 10 experimental assays (see below). Thus, neither repair of the Cas9-cleaved DNA, nor spontaneous mutations resulted in viruses possessing a modified functional p30 gene. This indicates that the targeted region of p30 (amino acids 71 to 78) is critical for function.

However, in two out of ten passage experiments of ASFV-BA71VΔTKdsRed infected WSL-gRp30 cells, plaque-forming virus could be reisolated, and PCR amplification and sequencing of the targeted p30 gene region revealed one or two nucleotide exchanges leading to one or two amino acid substitutions (Fig. 1C). Furthermore, several natural ASFV isolates from Kenya, Uganda, Tanzania and Burundi exhibit four base substitutions within the target region of the p30 gene which are identical to those found in the Kenyan virus used in this study (Fig. 1B). Three of them affect wobble bases at third codon positions and are translationally silent. The last one however, although also located at the third position of the codon, leads to a non-conservative amino acid substitution of histidine (H) by glutamine (Q) (Fig. 1B). At present it is not clear whether these substitutions are functionally tolerated, or depend on compensatory alterations elsewhere in p30 or within interacting proteins of the corresponding ASFV strains or mutants.

## Discussion

In summary, our experiments provide the first proof-of-principle that ASFV replication can be suppressed in cells expressing a CRISPR/Cas9 system targeting an essential virus gene. This novel approach is important because of the absence of other prophylactic or therapeutic measures against this lethal porcine infection^1^, and can now be applied to create Cas9 and guide RNA-expressing pigs by transgenesis. Previous studies have already shown the feasibility of stable expression of antiviral CRISPR/Cas9 systems in plants^29^, as well as in the silkworm *Bombyx mori*^28^. Different transgenes encoding e.g. human proteins could be also permanently expressed in pigs during attempts to make them suitable as organ donors for xenotransplantation^30,45^. To this end, fetal swine fibroblasts were stably transfected with the desired constructs, tested for stable transgene expression *in vitro* and injected into enucleated oocytes. After fusion and activation the embryos were transferred into the uterus of synchronized sows, and the desired transgenic animals were born. In a similar manner, we will attempt to generate pigs expressing ASFV-specific CRISPR/Cas9 systems. Since swine fibroblasts are in general hardly infectable by ASFV (results not shown), CRISPR/Cas9-expressing transgenic animals have to be generated first, before their macrophages as the natural ASFV host cells^6^ can be tested for susceptibility. If they exhibit significantly reduced levels of ASFV replication upon *in vitro* infection, the respective animals will be tested for resistance by challenge infections with virulent ASFV.

Our data show that escape mutations within the targeted part of the ASFV p30 gene are infrequent, and that expression of the p30 gene-specific gRNA leads to an approx.10,000fold reduction of virus replication in WSL cells. Thus it appears likely that a similar effect in transgenic pigs would prevent fatal infection with a matching virus. Innate and adaptive immune responses should then be able to eliminate the virus before escape mutants accumulate to significant levels. Furthermore, only few virulent ASF viruses possessing an altered p30 gene have been detected in the past (see above). Future studies have to demonstrate, whether a modified guide RNA matching this altered p30 gene sequence specifically and efficiently inhibits replication of the respective viruses, and whether cell lines expressing Cas9 nuclease together with both gRNAs are resistant against all natural ASFV variants. Moreover, like in herpesvirus-infected cells^43^ simultaneous targeting of additional virus genes by the CRISPR/Cas9 system could further increase its efficacy, in particular against multiple ASFV strains and isolates. To achieve this, we have started to adapt a multiplex CRISPR/Cas9 vector system^46^.

In modified approaches, the CRISPR/Cas9 system should also facilitate targeted deletion of CP204L and other essential or nonessential ASFV genes from the virus genome. This can be supported by generation of *trans*-complementing cell lines which express the affected proteins from codon-modified ORFs. Such ASFV recombinants could assist in elucidation of gene functions, and might be also suitable as attenuated or single-cycle live vaccines.

## Methods

### Cells and viruses

The ASFV-permissive wild boar lung cell line WSL was cultivated in Iscove′s Modified Dulbecco’s Medium with Ham’s F-12 Nutrient Mix and 10% fetal bovine serum as described^35^. For stable introduction of CRISPR/Cas9 plasmids the K2 Transfection System (Biontex) was used. Two days after transfection the cells were trypsinised, seeded into 96 well plates and maintained in medium containing 500 µg/ml G418. Single resistant cell clones detected after 2 to 3 weeks were further propagated.

ASFV-BA71VΔTKdsRed was derived from the Vero cell-adapted strain BA71V^33^ by substitution of codons 7 to 137 of the viral thymidine kinase (TK) gene (K196R) by the dsRed ORF (recloned from plasmid pDsRed-Express, Clontech) under control of the ASFV major capsid protein (p72) promoter. The virus was isolated after transfection of WSL cells with a corresponding transfer plasmid and subsequent wild-type ASFV infection, followed by selection of TK-negative recombinants on TK-negative WSL cells in the presence of 25 µg/ml bromodeoxyuridine as described^35,36^. ASFV-Kenya1033ΔCD2vdsRed, was derived from ASFV-Kenya1033 (GenBank submission in preparation) by substitution of the CD2v ORF (EP402R) from codon 77 to the translational stop codon (386) by the same dsRed expression cassette, and repeated plaque purification of fluorescent cell foci after transfer plasmid transfection and virus infection. Furthermore, an Armenian ASFV isolate from 2007 was used in this study, which, like ASFV-Kenya1033, had been adapted by ≤10 serial passages to more efficient replication in WSL cells. Reporter gene expression by the respective virus recombinants was detected by fluorescence microscopy (Axio Vert. A1 with software ZEN 2, Zeiss).

### Construction of the CRISPR/Cas9 expression plasmid

The two NLS within the Cas9 ORF of vector pX330-U6-Chimeric_BB-CBh-hSpCas9^20^ were removed by site directed mutagenesis (QuikChange II XL kit, Agilent), using the complementary primer pairs C9ΔNLS1-MF/MR and C9ΔNLS2-MF/MR (Table 1). Furthermore, a neomycin resistance gene under control of the simian virus 40 (SV40) early promoter was inserted into the obtained plasmid. To this end, it was linearized by digestion with *Pci*I, and ligated with a 1661 bp AseI fragment of pcDNA3.1(+) (Thermo Fisher Scientific) after Klenow fill-in of the non-compatible single-stranded overhangs, resulting in plasmid pX330-ΔNLS1/2neoR. Finally, the overlapping complementary oligonucleotide primers gRp30B-F and gRp30B-R (Table 1) were hybridised and inserted into the *Bbs*I-digested vector, resulting in pX330-ΔNLS1/2neoR-gRp30.

**Table 1:** Oligonucleotide primers used in this study.

Name	Sequence	Position
C9ΔNLS1-MF	GACGATGACGATAAGATG/GACAAGAAGTACAGCATC	1305–1322/1371–1388 [1]
C9ΔNLS2-MF	CTCAGCTGGGAGGCGAC/TAAGAATTCCTAGAGCTC	5455–5471/5520–5537 [1]
gRp30-F	caccGCAAGGGTATACTGAACATC	125,144–125,163 (R) [2]
gRp30-R	aaacGATGTTCAGTATACCCTTGC	125,144–125,163 (R) [2]
X330gRR-F	ATGCTTACCGTAACTTGAAAG	181–201 [1]
X330gRR-R	ATTTGTCTGCAGAATTGGCG	405–424 (R) [1]
CP204L-PSF	CACAAGTTGTGTTTCATGC	125290–125308 (R) [2]
CP204L-PSR	TGAAGATCCACGGTTACCC	124670–124688 [2]

Primers C9ΔNLS1-MR and C9ΔNLSR-MR were reverse complementary to the respective forward primers, and slashes (/) indicate the positions of the deleted NLS encoding sequences. Nucleotides printed in lower case have been added for convenient cloning of the hybridised oligonucleotides. Nucleotide positions refer to the forward or reverse (R) strand of the sequence of [1] pX330-U6-Chimeric_BB-CBh-hSpCas9^20^, or of [2] ASFV Georgia^34^.

### DNA preparation, PCR, and sequence analyses

Total DNA of infected and uninfected WSL cells was prepared as described^47^. To confirm presence of the ASFV-specific guide RNA gene, a 246 bp fragment of genomic cell DNA was amplified by PCR using primers X330gRR-F and X330-gRR-R (Table 1) and Pfx DNA polymerase (Thermo Fisher Scientific), and sequenced with the same primers using the BigDye Terminator v1.1 cycle sequencing kit, and a 3130 Genetic Analyzer (Applied Biosystems). The targeted p30 gene region of the ASFV genome was analysed by PCR amplification and sequencing of a 639 bp fragment with primers CP204L-PSF and CP204L-PSR (Table 1). Results were evaluated with the Geneious^®^ software package in version 10.2.3 (Biomatters).

### Western blot analyses

Proteins of WSL and WSL-gRp30 cell lysates were separated by discontinuous sodium dodecyl sulfate-polyacrylamide gel electrophoresis (SDS-PAGE), transferred to nitrocellulose membranes, and blots were subsequently incubated as described^48^. The anti-FLAG M2 monoclonal antibody (Sigma-Aldrich), and the α-tubulin specific monoclonal antibody B-5-1-2 (Sigma-Aldrich) were used at dilutions of 1: 5,000 or 1: 20,000, respectively. Binding of peroxidase-conjugated anti-mouse IgG antibodies (Jackson ImmunoResearch) was detected by chemiluminescence (Clarity Western ECL Substrate, Bio-Rad), and recorded (VersaDoc 4000 MP, Bio-Rad).

### Virus replication studies

For determination of plating efficiencies WSL-gRp30 and parental WSL cells were grown overnight in microtiter plates, and inoculated in parallel with serial dilutions of ASFV-BA71VΔTKdsRed, ASFV-Armenia, ASFV-Kenya1033ΔCD2v dsRed, or ASFV-Kenya1033 by centrifugation for 1 h at 600 × g and 25 °C. Then the inoculum was replaced by semisolid medium containing 6 g/ml methyl cellulose, and incubation was continued at 37 °C. After 5 days plaques and foci of infected cells were counted to calculate infectious virus titres (PFU/ml). For determination of growth kinetics, WSL-gRp30 and normal WSL cells grown in microtiter plates were inoculated as above at a MOI of 0.03 with ASFV-BA71VΔTKdsRed or ASFV-Kenya1033ΔCD2vdsRed. After removal of the inoculum, the cells were washed and incubated under normal medium at 37 °C. Immediately thereafter, and after 24, 36, 48, 72, 96, 144, and 192 h single plates containing cells plus medium were frozen at −80 °C. Finally, the cells were thawed at 37 °C and virus progenies in total cell lysates were analysed by titration on normal WSL cells.

### Biosafety

The Friedrich-Loeffler-Institut is licensed by the competent German authority to work with African swine fever virus, and all experiments with infectious ASFV were performed in improved biosafety level (BSL)−3 laboratories, which fulfill BSL-4 standards with respect to air filtration and effluent treatment.

### Data availability

All relevant data generated or analysed during this study are included in this published article.

## Electronic supplementary material

Supplementary figures

## References

[1] Galindo, I. & Alonso, C. African swine fever virus: A review. *Viruses* **9**, 10.3390/v9050103 (2017). 10.3390/v9050103 PMC5454416 28489063

[2] Guinat C (2016). Transmission routes of African swine fever virus to domestic pigs: current knowledge and future research directions. Vet Rec.

[3] ADNS. *ADNS disease overview*, https://ec.europa.eu/food/sites/food/files/animals/docs/ad_adns_outbreaks-per-disease.pdf (2017).

[4] King K (2011). Protection of European domestic pigs from virulent African isolates of African swine fever virus by experimental immunisation. Vaccine.

[5] Dixon LK, Chapman DA, Netherton CL, Upton C (2013). African swine fever virus replication and genomics. Virus research.

[6] Alcami A, Carrascosa A, Vinuela E (1990). Interaction of African swine fever virus with macrophages. Virus research.

[7] Kleiboeker SB, Scoles GA, Burrage TG, Sur JH (1999). African swine fever virus replication in the midgut epithelium is required for infection of ornithodoros ticks. Journal of virology.

[8] Costard S, Mur L, Lubroth J, Sanchez-Vizcaino JM, Pfeiffer DU (2013). Epidemiology of African swine fever virus. Virus research.

[9] Jori F (2013). Review of the sylvatic cycle of African swine fever in sub-Saharan Africa and the Indian ocean. Virus research.

[10] Lacasta A (2014). Expression library immunization can confer protection against lethal challenge with African swine fever virus. Journal of virology.

[11] Lokhandwala S (2017). Adenovirus-vectored novel African swine fever virus antigens elicit robust immune responses in swine. PLoS One.

[12] O’Donnell V (2017). Simultaneous deletion of the 9GL and UK genes from the African swine fever virus Georgia 2007 isolate offers increased safety and protection against homologous challenge. Journal of virology.

[13] Reis AL (2016). Deletion of African swine fever virus interferon inhibitors from the genome of a virulent isolate reduces virulence in domestic pigs and induces a protective response. Vaccine.

[14] Monteagudo, P. L. *et al*. BA71DeltaCD2: A new recombinant live attenuated African swine fever virus with cross-protective capabilities. *Journal of virology*, 10.1128/JVI.01058-17 (2017). 10.1128/JVI.01058-17 PMC5640839 28814514

[15] Freitas FB, Frouco G, Martins C, Leitao A, Ferreira F (2016). *In vitro* inhibition of African swine fever virus-topoisomerase II disrupts viral replication. Antiviral research.

[16] Keita D, Heath L, Albina E (2010). Control of African swine fever virus replication by small interfering RNA targeting the A151R and VP72 genes. Antiviral therapy.

[17] Horvath P, Barrangou R (2010). CRISPR/Cas, the immune system of bacteria and archaea. Science.

[18] Wiedenheft B, Sternberg SH, Doudna JA (2012). RNA-guided genetic silencing systems in bacteria and archaea. Nature.

[19] Cho SW, Kim S, Kim JM, Kim JS (2013). Targeted genome engineering in human cells with the Cas9 RNA-guided endonuclease. Nature biotechnology.

[20] Cong L (2013). Multiplex genome engineering using CRISPR/Cas systems. Science.

[21] Mali P (2013). RNA-guided human genome engineering via Cas9. Science.

[22] Soppe JA, Lebbink RJ (2017). Antiviral goes viral: Harnessing CRISPR/Cas9 to combat viruses in humans. Trends Microbiol.

[23] Hou P (2015). Genome editing of CXCR4 by CRISPR/cas9 confers cells resistant to HIV-1 infection. Sci Rep.

[24] Kang H (2015). CCR5 disruption in induced pluripotent stem cells using CRISPR/Cas9 provides selective resistance of immune cells to CCR5-tropic HIV-1virus. Mol Ther Nucleic Acids.

[25] Whitworth KM (2016). Gene-edited pigs are protected from porcine reproductive and respiratory syndrome virus. Nature biotechnology.

[26] Burkard C (2017). Precision engineering for PRRSV resistance in pigs: Macrophages from genome edited pigs lacking CD163 SRCR5 domain are fully resistant to both PRRSV genotypes while maintaining biological function. PLoS pathogens.

[27] Popescu L (2016). Genetically edited pigs lacking CD163 show no resistance following infection with the African swine fever virus isolate, Georgia 2007/1. Virology.

[28] Chen S (2017). Transgenic clustered regularly interspaced short palindromic repeat/Cas9-mediated viral gene targeting for antiviral therapy of Bombyx mori nucleopolyhedrovirus. Journal of virology.

[29] Zaidi SS, Tashkandi M, Mansoor S, Mahfouz MM (2016). Engineering plant immunity: Using CRISPR/Cas9 to generate virus resistance. Front Plant Sci.

[30] Petersen B, Niemann H (2015). Molecular scissors and their application in genetically modified farm animals. Transgenic Res.

[31] Ryu, J. & Lee, K. In *Methods in Molecular Biology* Vol. 1605 (ed Lee K.) Ch. Zygotic Genome Activation, 231–244 (Humana Press, 2017).

[32] Yuan M (2015). Efficiently editing the vaccinia virus genome by using the CRISPR-Cas9 system. Journal of virology.

[33] Yanez RJ (1995). Analysis of the complete nucleotide sequence of African swine fever virus. Virology.

[34] Chapman DA (2011). Genomic analysis of highly virulent Georgia 2007/1 isolate of African swine fever virus. Emerging infectious diseases.

[35] Portugal R, Martins C, Keil GM (2012). Novel approach for the generation of recombinant African swine fever virus from a field isolate using GFP expression and 5-bromo-2′-deoxyuridine selection. J Virol Methods.

[36] Keil GM, Giesow K (2014). & Portugal, R. A novel bromodeoxyuridine-resistant wild boar lung cell line facilitates generation of African swine fever virus recombinants. Archives of virology.

[37] Garcia-Escudero R, Vinuela E (2000). Structure of African swine fever virus late promoters: Requirement of a TATA sequence at the initiation region. Journal of virology.

[38] Rodriguez JM, Salas ML (2013). African swine fever virus transcription. Virus research.

[39] Afonso CL (1992). Characterization ofp30, a highly antigenic membrane and secreted protein of African swine fever virus. Virology.

[40] Prados FJ, Vinuela E, Alcami A (1993). Sequence and characterization of the major early phosphoprotein p32 of African swine fever virus. Journal of virology.

[41] Gomez-Puertas P (1998). The African swine fever virus proteins p54 and p30 are involved in two distinct steps of virus attachment and both contribute to the antibody-mediated protective immune response. Virology.

[42] Portugal RS, Bauer A, Keil GM (2017). Selection of differently temporally regulated African swine fever virus promoters with variable expression activities and their application for transient and recombinant virus mediated gene expression. Virology.

[43] van Diemen FR (2016). CRISPR/Cas9-mediated genome editing of herpesviruses limits productive and latent infections. PLoS pathogens.

[44] Lebbink RJ (2017). A combinational CRISPR/Cas9 gene-editing approach can halt HIV replication and prevent viral escape. Sci Rep.

[45] Niemann H, Petersen B (2016). The production of multi-transgenic pigs: update and perspectives for xenotransplantation. Transgenic Res.

[46] Sakuma T, Nishikawa A, Kume S, Chayama K, Yamamoto T (2014). Multiplex genome engineering in human cells using all-in-one CRISPR/Cas9 vector system. Sci Rep.

[47] Fuchs W, Mettenleiter TC (1996). DNA sequence and transcriptional analysis of the UL1 to UL5 gene cluster of infectious laryngotracheitis virus. The Journal of general virology.

[48] Pavlova SP, Veits J, Mettenleiter TC, Fuchs W (2009). Live vaccination with an H5-hemagglutinin-expressing infectious laryngotracheitis virus recombinant protects chickens against different highly pathogenic avian influenza viruses of the H5 subtype. Vaccine.

