# Supplementary material for: Efficient inhibition of African swine fever virus replication by CRISPR/Cas9 targeting of the viral p30 gene (CP204L)
Source: Sci Rep. 2018 Jan 23;8:1449. doi: 10.1038/s41598-018-19626-1 (PMC5780455; doi:10.1038/s41598-018-19626-1)
Supplement: Supplementary file 1 — Supplementary figures [file 41598_2018_19626_MOESM1_ESM.pdf]

Supplementary information to SREP-17-36383

Efficient inhibition of African swine fever virus replication by CRISPR/Cas9 targeting of the viral p30 gene (CP204L)

Alexandra Hübner, Bjoern Petersen, Günther M. Keil, Heiner Niemann, Thomas C. Mettenleiter, and Walter Fuchs
Friedrich-Loeffler-Institut, Greifswald – Insel Riems, Germany

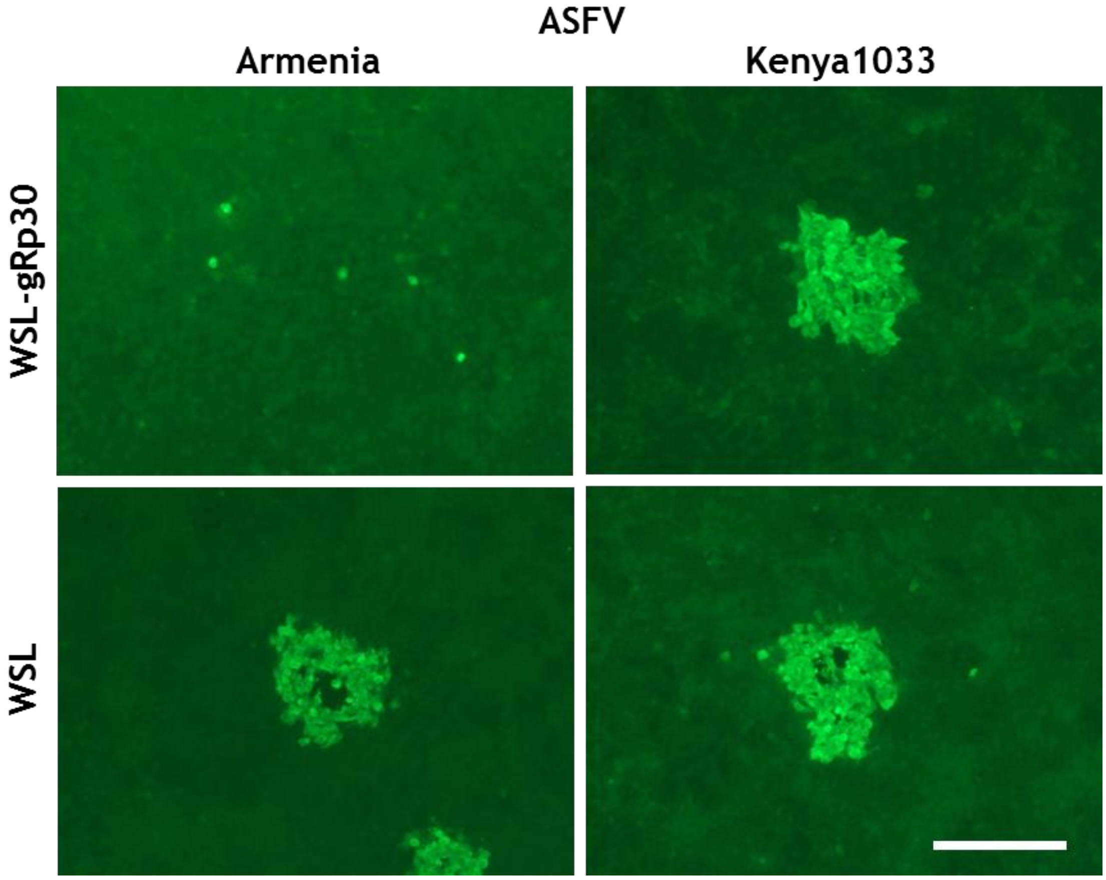

Figure S1. Indirect immunofluorescence analyses of WSL-gRp30 and WSL cell monolayers infected with ASFV-Armenia or ASFV-Kenya1033. The cells were fixed 5 days after infection with methanol-acetone (1:1), and subsequently incubated with a monospecific rabbit antiserum raised against a bacterially expressed fragment of the ASFV capsid protein p72 (amino acids 1 to 388), and Alexa-Fluor 488-conjugated anti-rabbit IgG (Thermo Fisher Scientific). Bar indicates 200 µm.

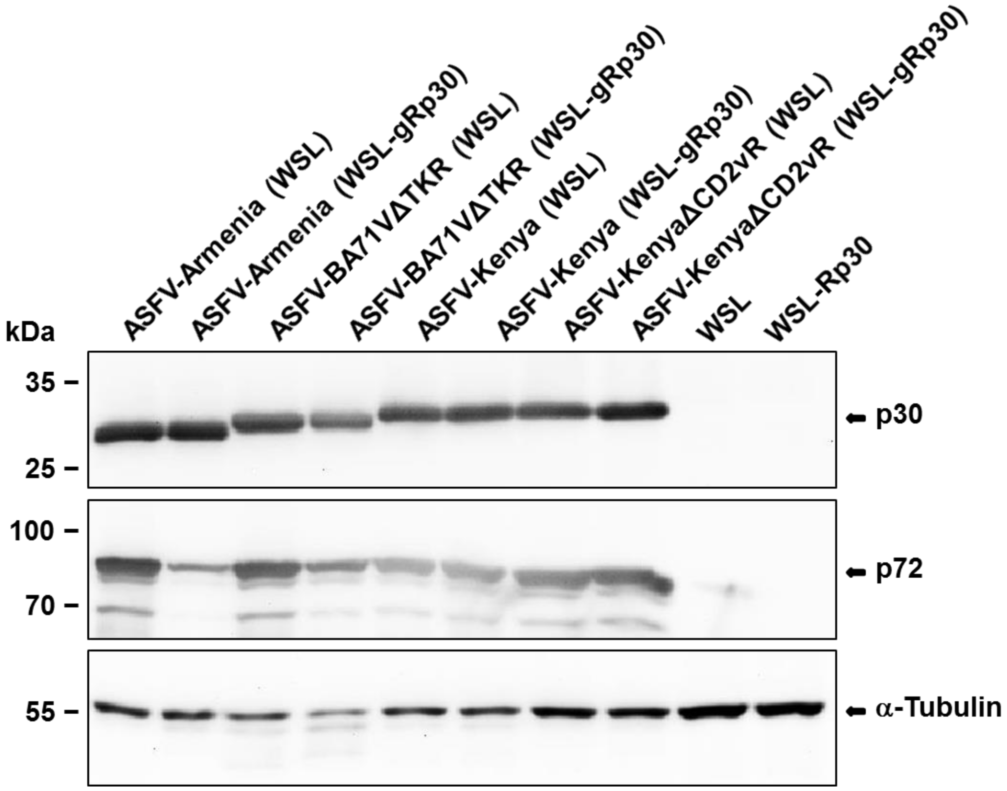

Figure S2. Western blot analyses of WSL-gRp30 and WSL cell lysates harvested 48 h after infection with ASFV-Armenia, ASFV-BA71VΔTKdsRed, ASFV-Kenya1033 or ASFV-Kenya1033ΔCD2vdsRed at a MOI of 5. Identical volumes (approx. 10^4 cells/lane) were separated on SDS polyacrylamide gels, and parallel blots were incubated with monospecific rabbit antisera raised against bacterially expressed ASFV p30 or p72, or a monoclonal antibody against α-tubulin as loading control (Sigma-Aldrich). Antibody binding was detected by chemilumescence reactions of peroxidase-conjugated anti-rabbit or anti-mouse IgG (Jackson Immunoresearch). Molecular masses of marker proteins are indicated.
